# *Cryptosporidium* spp. diagnosis and research in the 21^st^ century

**DOI:** 10.1016/j.fawpar.2021.e00131

**Published:** 2021-08-20

**Authors:** Jennifer K. O'Leary, Roy D. Sleator, Brigid Lucey

**Affiliations:** Department of Biological Sciences, Munster Technological University, Bishopstown Campus, Cork, Ireland

**Keywords:** *Cryptosporidium*, *Cryptosporidium parvum*, *Cryptosporidium hominis*, Infectious disease, Clinical diagnosis, CRISPR/Cas

## Abstract

The protozoan parasite *Cryptosporidium* has emerged as a leading cause of diarrhoeal illness worldwide, posing a significant threat to young children and immunocompromised patients. While endemic in the vast majority of developing countries, *Cryptosporidium* also has the potential to cause waterborne epidemics and large scale outbreaks in both developing and developed nations. Anthroponontic and zoonotic transmission routes are well defined, with the ingestion of faecally contaminated food and water supplies a common source of infection. Microscopy, the current diagnostic mainstay, is considered by many to be suboptimal. This has prompted a shift towards alternative diagnostic techniques in the advent of the molecular era. Molecular methods, particularly PCR, are gaining traction in a diagnostic capacity over microscopy in the diagnosis of cryptosporidiosis, given the laborious and often tedious nature of the latter. Until now, developments in the field of *Cryptosporidium* detection and research have been somewhat hampered by the intractable nature of this parasite. However, recent advances in the field have taken the tentative first steps towards bringing *Cryptosporidium* research into the 21^st^ century. Herein, we provide a review of these advances.

## Highlights

• *Cryptosporidium*, a protozoan parasite, is a leading cause of gastroenteritis worldwide.
• Despite its prevalence and impact, it is underreported, with no vaccine and few treatment options.
• While molecular diagnosis options are increasing, microscopy remains the gold standard.
• Many areas of research have also been limited by the intractable nature of this pathogen.
• Advances in molecular biology, e.g. NGS and CRISPR/Cas, stand to advance research in this field.

## Graphical abstract

**Figure:**
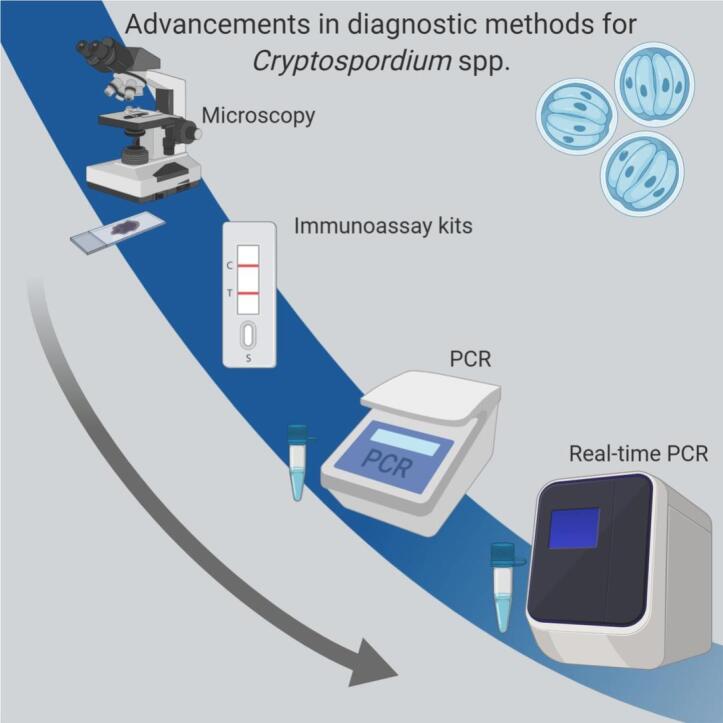

## 1 Introduction

*Cryptosporidium* is an obligate enteric protozoan parasite and a well-established pathogen of the gastrointestinal tract (Tzipori and Widmer, 2008). Originally described from histological preparations of murine gastric mucosa in 1907 (Tyzzer, 1910), *Cryptosporidium* was not linked with human infection until 1976 (Nime et al., 1976). Sentinel alert of the clinical significance of cryptosporidial infection was established after reports of fatal cryptosporidiosis in AIDS patients during the 1980s (Soave et al., 1984), and a large waterborne outbreak affecting approximately 400,000 Milwaukee residents in 1993 (D’Antonio et al., 1985; MacKenzie et al., 1995; Soave et al., 1984). *Cryptosporidium* is environmentally and geographically ubiquitous, comprising many species spanning a wide host range. To date over 40 species have been described, with at least 20 species having been reported in human infection (Feng et al., 2018; Xiao and Feng, 2017). However, *C. hominis* and *C. parvum* have been reported to account for the vast majority of infections in humans (Feng et al., 2018).

*Cryptosporidium* has now emerged as a leading cause of diarrhoeal illness worldwide, posing a significant threat to young children and immunocompromised patients. It has been reported to be a leading cause of moderate-to-severe gastrointestinal morbidity in children younger than 5 years in developing countries (Kotloff et al., 2013). A recent study into the global burden of gastrointestinal disease found that *Cryptosporidium* spp. accounted for in excess of 1 million deaths, almost half a million of which were in children under the age of five, and over 71 million disability-adjusted life years (DALYs) between 2005 and 2015; the highest mortality rates were observed in developing countries, particularly those in sub-Saharan Africa (Troeger et al., 2017).

While endemic in the vast majority of developing countries, *Cryptosporidium* also has the potential to cause waterborne epidemics and large scale outbreaks in both developing and developed nations (Efstratiou et al., 2017). Anthroponontic and zoonotic transmission routes are well defined, with the ingestion of faecally contaminated food and water supplies a common source of infection (King et al., 2019; McKerr et al., 2019; Ryan et al., 2018).

Microscopy, the current diagnostic mainstay, is considered by many to be suboptimal. This has prompted a shift towards alternative diagnostic techniques in the advent of the molecular era. Molecular methods, particularly PCR, are gaining traction in a diagnostic capacity over microscopy in the detection of *Cryptosporidium* spp., given the laborious and tedious nature of the latter. The supersession of microscopy by molecular techniques, which offer rapid diagnosis and improved sensitivity, may also be attributed to diminishing microscopy skills in modern clinical laboratories (Meurs et al., 2017). Until now, developments in the field of *Cryptosporidium* detection and research have been somewhat hampered by the intractable nature of this parasite. However, recent advances, particularly in the application of the CRISPR/Cas system to produce genetically modified, tractable *Cryptosporidium* oocysts (Vinayak et al., 2015), have allowed the field to take tentative steps towards bringing *Cryptosporidium* research into the 21^st^ century.

## 2 Diagnosis

Given the broad spectrum of susceptibility and the significant morbidity and mortality rates associated with *Cryptosporidium* in immunosuppressed patient populations, the development of efficient and effective screening criteria and robust testing algorithms is vital in clinical laboratories (Bruijnesteijn van Coppenraet et al., 2009; Garcia et al., 2003; Hawash, 2014). However, there remains no international standard methods for the diagnosis of cryptosporidiosis. In some countries *Cryptosporidium* testing is limited to known HIV/AIDS patients, however, testing of adult samples is usually reliant upon stipulating factors such as watery or persistent diarrhoea, and when clinically suspected (Chalmers, 2008). Current UK microbiological standards recommend that all symptomatic cases of acute diarrhoea are investigated based on guidelines for faecal screening for *Cryptosporidium*, published in 1993 (Casemore and Roberts, 1993). These guidelines are the culmination of a two year prospective survey conducted in 16 clinical laboratories on some 62,000 patients, which provided data pertaining to the absolute and relative frequency of *Cryptosporidium* infection across all age groups and consequently allowed for meaningful age-based selection criteria to be determined (Casemore and Roberts, 1993; Palmer and Biffin, 1990; Public Health England, 2014). Compliance with these guidelines is not absolute, several UK-based studies determined that routine screening of all diagnostic stool specimens for *Cryptosporidium* in participating clinical laboratories ranged from 33 to 72.5% (Chalmers and Thomas, 2002; Chalmers et al., 2015). The more recent of these studies found that 27.5% of surveyed laboratories conducted for *Cryptosporidium* spp. testing on samples based on one or more selection criteria, including stool consistency (19% [16 of 85]), patient age (21 % [18 of 85]), history or clinical details (47 % [40 of 85]), duration of hospitalization (21% [18 of 85]), or clinician requests (29 % [25 of 85]), prompting further revision of UK national standards to encourage testing of all submitted samples (Chalmers et al., 2015).

Owing to higher prevalence of *Cryptosporidium* in children, patient age is currently used as the primary selection criterion for *Cryptosporidium* testing within the UK and Ireland (Chalmers and Davies, 2010). It is widely acknowledged that age bias impacts on the reported age distribution rates of a variety of pathogens, including *Cryptosporidium*. Adding to this bias are parental and health care professional behaviours towards gastrointestinal symptoms and stool samples collection, respectively, leading to higher reporting of *Cryptosporidium* cases in children than in adults (Garvey and McKeown, 2009). There also remains a wide-scale lack of standardisation in clinical *Cryptosporidium* detection practices both within and between nations, and comparative information is particularly limited (Manser et al., 2014).

In Ireland, a 2004 report by the Health Protection Surveillance Centre in Ireland (HPSC) recommended the testing of all stool samples from patients exhibiting clinical symptoms associated with cryptosporidiosis (Health Protection Surveillance Centre, 2004). However, should resource and logistical constraints prohibit this, it is recommended that all patients under the age of 10 be screened as an alternative, with a comparable age threshold of 15 advised in a similar UK report (Crook et al., 2002). It is important to note, however, that should such an age threshold be employed, it should only be applied for the investigation of sporadic cases rather than in outbreak situations. A study of a 2007 outbreak in Galway, in the Republic of Ireland concluded that 40% of all infections occurred in patients over the age of 15. Thus, it appears likely that laboratories using such thresholds also fail to detect a large proportion of sporadic cases (Pelly et al., 2007)**.**

In many countries, such as the United States and France, *Cryptosporidium* screening is not a routine component of standard “ova plus parasite” examinations carried out in clinical laboratories regardless of patient age, clinical and epidemiological evidence, unless specifically requesting by a clinician or recommended by the laboratory directorate. Thus, further contributing to the under reporting of cases (ANOFEL Cryptosporidium National Network, 2010; Chen et al., 2002). Meanwhile, a recent European survey of 18 laboratories found noteworthy variety between detection methods with almost all laboratories relying upon microscopic methods either alone or in combination with other detection methods. Half of all laboratories surveyed used at least two methods of detection, only two of which employed molecular diagnostics (Manser et al., 2014).

Expertise in the field of stool microscopy is declining among the modern clinical laboratory workforce, particularly in areas of decreasing prevalence of faecal parasites (McHardy et al., 2014). While a number of studies have reported that molecular methods are employed by only a minority of routine clinical microbiology laboratories in Europe and the US (Chalmers and Davies, 2010; Fournet et al., 2013; Jones et al., 2004; Manser et al., 2014; ten Hove et al., 2007), the growing acceptance of molecular methods and increasing throughput of samples has prompted the necessity for automated, walk-away technology in these laboratories.

Additionally, given the low prevalence of *Cryptosporidium* within the population in developed countries at least, certain laboratories may not receive adequate levels of positive samples to establish and maintain expertise in this area (McHardy et al., 2014). Consequently, although microscopy is currently regarded as the gold standard in the diagnosis of cryptosporidiosis, it seems likely that molecular methods will eventually replace microscopy altogether.

A summary of the advantages and disadvantages associated with the various diagnostic methods discussed herein are outlined in Table 1.

**Table 1:** Advantages and disadvantages of microscopic, immunological and molecular diagnostic methods for *Cryptosporidium* spp.

Diagnostic Test	Advantages	Disadvantages
Microscopy	• Relatively low cost • Widely available	• Poor sensitivity • Time consuming • Skilled microscopist essential
Immunoassay based methods	• Good sensitivty • Wide variety of kits available • Convenient adjunct to microscopic analysis	• Not widely available in developing countries due to cost constraints and limited detection spectrum of kits • False positives
Molecular/Nucleic acid amplification methods	• Exceptional sensitivity • Capable of species and subspecies identification • Option to mulitiplex detection of several enteric pathogens	• Expensive reagents and instrumentation required • Requires skilled technician

### 2.1 Brightfield and fluorescent microscopy

Faecal investigation for the presence of shed oocysts or antigens is the diagnostic mainstay in *Cryptosporidium* detection (Manser et al., 2014). Conventional clinical diagnosis has largely relied on microscopic examination of tinctorially or fluorescently stained faecal smears. The acid-fast properties of *Cryptosporidium* were demonstrated in 1981, with the development of a modified Ziehl-Neelson (mZN) stain for differential staining (Henricksen and Pohlenz, 1981). Prior to this *Cryptosporidium* was largely identified through Giemsa staining of histological preparation of intestinal biopsy samples, with iodine, trichrome and iron haematoxylin stained faecal specimens yielding poor results (Kissinger, 2008; McNabb et al., 1985).

A variety of stains including the acid-fast Kinyoun’s stain and differential stains such as the hot safrinin-methylene blue stain have also been employed by clinical laboratories (Baxby et al., 1984; Kageruka et al., 1984). In addition, a variety of negative and fluorescent stains have been developed. Both staining techniques provide a rapid, inexpensive, sensitive alternative to the acid-fast techniques (Casemore et al., 1985; Garcia et al., 1983; Hanscheid et al., 2008; Khurana et al., 2012; Vohra et al., 2012). Despite this, acid-fast staining, particularly the mZN technique, predominates in clinical laboratories (Manser et al., 2014). However, although there is a marked contrast between the red stained oocyst against the green background counterstain of the mZN, yeasts, fungal and bacterial spores may be erroneously identified as oocysts (Casemore, 1991). Intermittent oocyst shedding is inherent to the *Cryptosporidium* life cycle. Thus, in order to improve diagnostic sensitivity, faecal specimens are often collected over three different days, adding to the laboratory workload (Goñi et al., 2012; van Gool et al., 2003). Overall, this time consuming and tedious staining technique demands an experienced microscopist, but exhibits poor sensitivity (37-100%) (Abou El-Naga and Gaafar, 2014; Chalmers et al., 2011; Kaushik et al., 2008; Tuli et al., 2010; Zaglool et al., 2013).

Unsurprisingly, given the variable levels of sensitivity associated with brightfield and fluorescent staining techniques, a number of oocyst concentration methods have been developed in order to maximise oocyst yields from faecal samples (Garcia et al., 1983; Weber et al., 1991). These techniques are most useful when preserved stool specimens are received in epidemiological cases, asymptomatic cases, and in immunocompromised patients with a clinical history of unexplained diarrhoea, as this patient population is susceptible to recrudescence following periods of remission (Casemore, 1991; Omoruyi et al., 2014).

Although faecal staining methods remain the cornerstone of parasitological investigations in both American and European clinical laboratories, these methods were previously surpassed by a variety of immunological and most significantly, molecular identification methods (Jones et al., 2004; Manser et al., 2014). *Cryptosporidium* targeting, immunofluorescent monoclonal antibodies (MAb) were initially introduced almost three decades ago following the advent of hybridoma technology, which allowed for the generation of highly specific antibodies (Milstein, 1999; Sterling and Arrowood, 1986). Monoclonal oocyst wall antibodies have been conjugated with fluorescent labels such as fluorescein isothiocyanate (FITC) and biotin hydrazide, which imparts a distinct apple green-to-yellow fluorescence to the oocysts against a dark background, allowing visualisation of intact parasites (Arrowood and Sterling, 1989; Garcia et al., 1987; Sterling and Arrowood, 1986). Comparative studies have found the sensitivity and specificity of immunofluorescent techniques to outweigh the sensitivity and specificity exhibited by conventional brightfield staining techniques (Alles et al., 1995; Arrowood and Sterling, 1989; Current and Garcia, 1991; Elsafi et al., 2014; Garcia et al., 1992; Kamal et al., 2008). Additionally, indirect immunofluorescent techniques, although requiring an additional incubation step, have been reported to possess similar levels of sensitivity and specificity to those of their direct counterparts (Rusnak et al., 1989; Stibbs and Ongerth, 1986).

While immunofluorescent detection of *Cryptosporidium* spp*.* necessitates the use of a fluorescent microscope, which has prevented widespread utilisation of this method and may preclude the use of this technique in developing countries, the marked distinction of oocysts from the non-fluorescent background conveniently reduces the amount of time required for microscopic investigation (Vohra et al., 2012). In addition, faecal concentration is not a prerequisite when faecal samples contain a paucity of oocysts, given the sensitivity of this method (Elsafi et al., 2014). Immunofluorescence enhances the ease with which less experienced microscopists can definitively identify the presence of oocysts (Alles et al., 1995; Garcia et al., 1987).

### 2.2 Enzyme immunoassays (EIA), ELISA and immunochromatographic methods

Faecal-antigen diagnostic techniques have been developed in order to obviate the need for skilled microscopists, laborious methodologies and specialised equipment, such as fluorescent microscopes, while also accommodating batch testing requirements (Helmy et al., 2014; Rosenblatt and Sloan, 1993; Ungar, 1990). Indeed, the colorimetric principles underlying quantitative enzyme immunoassays (EIA) and enzyme linked immuno-sorbent assays (ELISA) eliminate the requirement for extensive microscopy training of lab personnel and subjectivity associated with conventional microscopy (Goñi et al., 2012; Newman et al., 1993). Comparative studies investigating the diagnostic utility of EIA and ELISA kits have found that they provide significantly improved sensitivity (94 - 100%) and specificity (93 -100%) over conventional acid-fast staining methods (Kehl et al., 1995; Parghi et al., 2014; Siddons et al., 1992). However, comparisons between fluorescent and immunofluorescent staining methods have indicated enzyme-based immunological detection of *Cryptosporidium* to be inferior, with reduced capabilities of detecting low oocyst densities (Johnston et al., 2003; Kehl et al., 1995; Newman et al., 1993; Weitzel et al., 2006). In addition, several cases detailing the generation of false positive results by the ProSpect *Cryptosporidium* immunoassay (Alexon, Inc., Mountain View, California) have been reported, perhaps owing to faecal antigen shedding often persisting after intact oocyst shedding has abated (Doing et al., 1999; Johnston et al., 2003; Miller and Mojica, 1999).

Immunochromatographic kits provide a detection system that surpasses enzyme-based methods in terms of rapidity by eliminating the need for additional reagent additions, washing steps and incubations (Current and Garcia, 1991; Garcia et al., 2003). Antigen migration *via* capillary action allows detection of *Cryptosporidium* antigens by a discrete, colloidal dye labelled antibody impregnated in a line assay, permitting objective antigen detection (Llorente et al., 2002). Sensitivities and specificities of these qualitative faecal-antigen kits vary considerably, with one study which investigated four kits reporting ranges of 47 - 71 % and 98 - 100%, respectively, when compared to microscopy (Agnamey et al., 2011). Meanwhile three independent studies reported sensitivities and specificities of 98% and 100%, 98% and 100%, 100% and 99%, respectively, in immunochromatographic kits. In each study the immunochromatographic kits in question were compared to microscopy, EIA and ELISA, respectively (Chan et al., 2000; Garcia and Shimizu, 2000; Youn et al., 2009).

Like their enzyme-based counterparts, immunochromatographic kits have been found to generate false positive results, resulting in one case, for example, in a lot recall of the CoulorPAC^TM^ *Cryptosporidium*/Giardia rapid assay kit (Haupst and Davis, 2002). Immunoassay based kits also offer a reduced diagnostic spectrum, as many are tailored solely for the detection of *C. parvum* and *C. hominis*. Therefore the clinical utility of such kits is limited in regions where alternative *Cryptosporidium* species are attributable to a significant number of cryptosporidiosis cases (Agnamey et al., 2011; Llorente et al., 2002). Consequently, despite the logistical and economical improvements in assay methodology over conventional staining methods, enzyme and non-enzyme based immunoassays are not deemed to be a suitable substitution for such techniques in the modern clinical laboratory, even though they may be used as a confirmatory adjunct to conventional methods in clinical laboratories with limited experience in *Cryptosporidium* detection, or for epidemiological studies (Checkley et al., 1997; Goñi et al., 2012; Hanson and Cartwright, 2001; Weitzel et al., 2006)***.***

### 2.3 Molecular approaches

Following its inception in 1983, the polymerase chain reaction (PCR) has vastly improved molecular diagnostic approaches in many fields, including clinical microbiology (Espy et al., 2006; Tong and Giffard, 2012). PCR detection of *Cryptosporidium* has been proven to be more sensitive than conventional microscopic and immunological methods, while also permitting batch testing, species and sub-species identification of detected organisms (Chalmers et al., 2011; Elsafi et al., 2013; Aghamolaie et al., 2016; Uppal et al., 2014).

A sequence survey identifying >250 kb of the *C. parvum* genome heralded the beginning of the genomic era of *Cryptosporidium* research in the late 1990s (Liu et al., 1999). Given the clinical significance and the dearth of epidemiological and molecular *Cryptosporidium* data at the time, the National Institute of Allergy and Infectious Diseases (NIAID) subsequently allocated funding to a consortium of three American universities, which were tasked with sequencing both *C. parvum* and *C. hominis* genomes (Widmer and Sullivan, 2012). Two separate studies, undertaking whole genome shotgun sequencing strategies, subsequently yielded the fully sequenced genomes of *C. parvum* IOWA and *C. hominis* TU502 isolates (Abrahamsen et al., 2004; Xu et al., 2004)*.*This research ultimately led to the establishment of CryptoDB in 2003, an online database of known *Cryptosporidium* genomes (Heiges et al., 2006; Puiu et al., 2004). This collaborative effort integrates all genomic and functional genomic data pertaining to *Cryptosporidium* spp. in a single online, bioinformatics resource. At present, CryptoDB houses 15 genome sequences encompassing nine different species/genotypes, while 52 genome assemblies are available for the *Cryptosporidium* genus in the NCBI GenBank (Baptista et al., 2021). These genomic advances have paved the way for current epidemiological studies, functional analyses, protein and metabolic pathway predictions and genome annotation (Isaza et al., 2015; Mazurie et al., 2013).

In the initial advent of molecular techniques, characterisation of *Cryptosporidium* genotypes was largely achieved through PCR-mediated amplification of specific genetic regions, followed by enzymatic cleavage or sequencing (Cheun et al., 2013; Ibrahim et al., 2021; Insulander et al., 2013; Sulaiman et al., 2005). Prior to the *C. hominis* and *C. parvum* whole genome sequencing projects and the widespread availability of DNA sequencing techniques, RFLP was the primary means by which to conduct inter-species genotyping on *Cryptosporidium* spp. (Awad-el-Kariem et al., 1994; Leng et al., 1996). This technique utilises a number of key molecular markers amenable to PCR amplification and restriction digestion to produce unique banding patterns that are visualised *via* gel electrophoresis (Roellig and Xiao, 2020). However, RFLP based genotyping is limited in that it cannot resolve differences between isolates of the same genotype, particularly *C. hominis* and *C. parvum,* necessitating an alternative sub-genotyping technique (Roellig and Xiao, 2020)*.*

The *C. parvum* and *C. hominis* genome sequencing projects enabled the identification of a number of highly polymorphic micro- and minisatellite loci and conserved loci flanking sequences (Aiello et al., 1999; Cacciò et al., 2000; Feng et al., 2000). These sequences permitted the development of microsatellite and minisatellite locus-specific PCR assays for both species regions, thereby permitting genotyping superior to that of RFLP, and ultimately the development of a technique that is also capable of identifying the subtleties of intra-species differentiation (Ramo et al., 2015; Xiao and Feng, 2017).

Key molecular markers for species identification include the small sub-unit rRNA (SSU rRNA), *Cryptosporidium* outer wall protein (COWP), 70-kDa heat shock protein (HSP70), thrombospondin-related adhesive protein (TRAP-C2) and actin genes (Roellig and Xiao, 2020; Elwin et al., 2013; Hadfield et al., 2011). These regions contain large numbers of interspecific polymorphisms, which make them ideal for basic species identification. Owing to low levels of intraspecific variation, the SSU rRNA gene is the most widely used of these genetic targets in genotypic differentiation between an array of human and animal infecting species (de Lucio et al., 2016; Roellig and Xiao, 2020). Highly variable regions, such as the tandem repeat containing 60-kDa glycoprotein (*gp60*) gene and the microsatellite loci, ML1 and ML2, are predominantly used for this purpose given the marked amount of intra-species sequence heterogeneity expressed in these regions (Robinson and Chalmers, 2012). These regions have been pivotal in the determination of the extensive number of *C. parvum* and *C. hominis* sub-species. Extensive panels including these markers, among many others, are commonly employed in multi-locus sequence typing (MLST) based epidemiological studies to identify population structures, and inter- and intra-species genetic diversity (De Waele et al., 2013; Feng et al., 2014; Garcia-R et al., 2020; Wang et al., 2015; Xiao, 2010).

Within the *Cryptosporidium* genus and more specifically among the predominant human-pathogenic species, *C. parvum* and *C. hominis*, asexual and sexual life cycle stages, genetic recombination and selective pressures, such as parasite-host coevolution, host adaptation and geographic segregation, have led to generation of new subtype families and diverse genetic populations (Abal-Fabeiro et al., 2013; Feng et al., 2002; Garcia-R and Hayman, 2017). *gp60,* which is firmly established as a key marker of genetic variation within *Cryptosporidium* spp (Abal-Fabeiro et al., 2013), is subject to selective pressure which has resulted in a lack of global sub-structuring, with the same *gp60* alleles emerging in different locations globally (Abal-Fabeiro et al., 2013; Widmer, 2009). Thus, *gp60* is not a sufficient descriptor of population structure to enable single locus typing (Robinson and Chalmers, 2012). Multi-locus genotyping (MLG) is necessary to adequately assess genetic variation and population structures within *Cryptosporidium* spp.

Research is ongoing into the identification of novel genetic markers, and refining known markers into an internationally standardised MLG scheme (Chalmers et al., 2018). Population-level analyses (nucleotide diversity (*θ*π) and Watterson’s theta (*θ*_W_), Tajima’s D statistic etc.) have been used in several studies seeking to assess genetic diversity and evolutionary processes at multiple loci within *Cryptosporidium* spp. in order to aid understanding of host-parasite adaptation and evolution (Garcia-R et al., 2020; Garcia-R and Hayman, 2017). MLG, accomplished *via* DNA sequencing of PCR amplified amplicons from specific loci, or real-time PCR based high resolution melting analysis of loci amplicons, is also being evaluated for epidemiological surveillance and outbreak investigations in clinical laboratories (Chalmers et al., 2017; O'Leary et al., 2021a, O'Leary et al., 2021b).

Overall, several studies have recommended the incorporation of PCR techniques into routine clinical *Cryptosporidium* diagnosis methods (Rubio et al., 2014; Stensvold et al., 2011; Uppal et al., 2014). Indeed, PCR has been reported as having improved sensitivity over current detection methods, with reported limits of detection ranging from 1 × 10^5^ to 1 oocyst/Gram of faeces (Costa et al., 2021). This is a significant improvement in detection when compared to the limit of detection associated microscopy based detection, which requires between 1 × 10^4^ – 5 × 10^4^ oocysts per mL of faeces (Khurana and Chaudhary, 2018). Although promising results have been reported, standard PCR techniques, particularly nested PCR, are not ideally suited to routine human diagnostics as they have been associated with considerable contamination risks, owing to multiple rounds of DNA amplification and concomitant DNA manipulation steps.

The development of real-time PCR (or quantitative PCR, qPCR) offers a convenient alternative to conventional techniques. Completed within an hour or less, qPCR reaction times are superior to those seen in conventional PCR methods (Espy et al., 2006). Fluorescent probes are used to detect DNA amplification, while the closed reaction vessel ensures that contamination is comparably negligible to that associated with conventional PCR (Espy et al., 2006; Minarovičová et al., 2009). In addition, sensitivity and specificity levels equal to, or surpassing, those observed in conventional PCR have been reported (Elsafi et al., 2013; Hadfield et al., 2011; Liu et al., 2013). Table 2 provides a comparison between reported sensitivities and specificities of common microscopic methods versus currently available commercial immunological and DNA-based diagnostic panels.

**Table 2:** Sensitivities and specificities of microscopic methods, and currently available immunological and DNA-based diagnostic tests for *Cryptosporidium* spp.

Microscopic Staining Techniques			
Test Name	Sensitivity	Specificity	Reference
mZiehl-Neelsen stain	37–79.1%	100%	Chalmers et al. (2011); Kaushik et al. (2008); Khurana et al. (2012)
Fluorescent – auramine phenol stain	92.1 –100%	99.6–100%	Abou El-Naga and Gaafar (2014); Chalmers et al. (2011); Khurana et al. (2012)
Kinyoun’s acid fast stain	66.7–91.6%	88.2–100%	Abou El-Naga and Gaafar (2014); Elsafi et al. (2014)


Commercial Immunological Diagnostic Tests for *Cryptosporidium* spp.					
Test Name	Sensitivity^a^	Specificity^a^	Additional Pathogens Detected	Supplier	Reference
Crypto-Strip	47.2%	100%	N/A	Coris BioConcept, Gembloux, Belgium	Agnamey et al. (2011)
*Cryptosporidium* and *Giardia* Duo-Strip	91.7%	100%	*Giardia duodenalis*	Coris BioConcept, Gembloux, Belgium	Van den Bossche et al. (2015)
*Cryptosporidium* EZ VUE lateral-flow test strips	89%	99%	N/A	TechLab Inc., Blacksburg, Virginia, United States	Johansen et al. (2021)
*Cryptosporidium* II test	71.8%	94.3%	N/A	TechLab Inc., Blacksburg, Virginia, United States	Kabir et al. (2018)
*Giardia*/*Cryptosporidium* Quik Chek	92.3–100%	97.1–100%	*G. duodenalis*	TechLab Inc., Blacksburg, Virginia, United States	Chalmers et al. (2011); Van den Bossche et al. (2015); Kabir et al. (2018); Minak et al. (2012)
ImmunocardSTAT® C/ G	5.5–96%	96.6–100%	*G. duodenalis*	Meridian Bioscience Inc., Cincinnati, Ohio, United States	Agnamey et al. (2011); El-Moamly and El-Sweify (2012); Bouyou-Akotet et al. (2016)
ImmunoCard STAT!® CGE	100%	45.6–100%	*G. duodenalis, and Entamoeba histolytica*	Meridian Bioscience Inc., Cincinnati, Ohio, United States	Van den Bossche et al. (2015)
RIDA®QUICK	62.4–84.9%	98%	N/A	R-Biopharm Diagnostic, Darmstadt, Germany	Agnamey et al. (2011); Chalmers et al. (2011)
RIDA®QUICK Combi	60.4–100%	100%	*G. duodenalis, and E. histolytica /dispar*	R-Biopharm Diagnostic, Darmstadt, Germany	Van den Bossche et al. (2015); Helmy et al. (2014)
Remel ProSpecT	91.4%	100%	*G. duodenalis*	ThermoScientific, Waltham, Massachusetts, United States	Chalmers et al. (2011)
Remel-Xpect	68.8%	100%	N/A	ThermoScientific, Waltham, Massachusetts, United States	Agnamey et al. (2011)


Commercial Molecular Diagnostic Tests for *Cryptosporidium* spp,					
Test Name	Sensitivity^a^	Specificity^a^	Additional Pathogens Detected	Supplier	Reference
Allplex^TM^ Gastrointestinal Panel-Parasite Assay (GIPPA)	100%	Up to 100% – further testing needed.	*G. duodenalis*, *E. histolytica*, *Dientamoeba fragilis*, *B. hominis*, and *Cyclospora cayetanensis*	Seegene Inc, Seoul, Korea	Autier et al. (2020); Paulos et al. (2019)
Amplidiag® Stool Parasites	10^3^ oocysts/g	Not specified	*G. duodenalis, E. histolytica*, and *D. fragilis*	Mobidiag, Espoo, Finland	Costa et al. (2021)
BD Max parasitic panel (EPP)	95.5%	99.6%	*G. duodenalis,* and *E. histolytica*	BD Diagnostics, Sparks, Maryland, United States	Madison-Antenucci et al. (2016); Mölling et al. (2016)
Biofire FilmArray^TM^ Gastrointestinal Panel	100%	99.6–100%	14 bacterial and 5 viral targets. 3 further parasites: *G. duodenalis*, *E. histolytica*, and *C. cayetanensis*	BioFire Diagnostics, Salt Lake City, Utah, United States	Murphy et al. (2017); Binnicker (2015); Khare et al. (2014); Zhang et al. (2015)
EasyScreen^TM^ Enteric Parasite Detection Kit	100%	100%	G*. duodenalis, Entamoeba* complex; *D. fragilis*, and *Blastocystis* spp.	Genetic Signatures, Sydney, Australia	Stark et al. (2014)
EntericBio GastroPanel II	100%	100%	4 bacterial targets and *G. duodenalis*	Serosep. Limerick, Ireland	McAuliffe et al. (2017)
FTD Stool Parasites	53.1%	Up to 100% – further testing needed.	*Giardia* spp, and *E. histolytica*	Fast Track Diagnostics, Esch-sur-Alzette, Luxembourg	Paulos et al. (2019)
Gastroenteritis/Parasite Panel I	92–100%	Up to 100% – further testing needed.	*G. duodenalis*, and *E. histolytica*	Diagnode, Seraing, Belgium	Paulos et al. (2019)
Luminex xTAG® Gastrointestinal Pathogen Panel	95–100%	100%	12 bacterial and viral targets and 2 further parasites: *G. duodenalis* and *E. histolytica*	Luminex Corporation, Austin, Texas, United States	Patel et al. (2014); Wessels et al. (2014); Claas et al. (2013); Navidad et al. (2013); Perry et al. (2014); Zhang et al. (2015)
NanoCHIP® Gastrointestinal Panel (GIP)	Detection limit of 5 x 10^3^ oocysts	Up to 100% – further testing needed.	3 bacterial targets and *G. duodenalis*, *E. histolytica*, *E. dispar*, *D. fragilis*, and *Blastocystis hominis*	Savyon Diagnostics, Ashdod, Israel	Dror et al. (2016)
ParaGENIE Crypto-Micro PCR	91.7%	100%	Also differentiates between *Enterocytozoon bieneusi* and *Encephalitozoon intestinalis*	Ademtech, Pessac, France	Morio et al. (2019)
RIDA®GENE Parasitic Stool Panel	87.5%	Up to 100% – further testing needed.	*G. duodenalis, E. histolytica,* and *D. fragilis*	R-Biopharm Diagnostic, Darmstadt, Germany	Paulos et al. (2019)
QIAStat Dx® GIP	Not specified	Not specified	14 bacterial targets, 6 viral targets and 3 further parasites: *G. lamblia, E. histolytica,* and *C. cayetanensis*	Qiagen, Hilden, Germany	Boers et al. (2020)

^a^Sensitivity and specificity for *Cryptosporidium* spp. only are given here. Variable sensitivities and specificities reported for other pathogens detection by these panels.

Multiplex PCR has become a popular means by which to investigate the presence of multiple enteric parasites in a single sample. Initial studies found multiplex qPCR assays to be both 100% sensitive and specific when compared to the monoplex qPCR assays, with superior sensitivity and specificity over conventional microscopic methods for each of the protozoan parasites tested (Stark et al., 2011). This technology continues to increase in popularity for use in clinical laboratories, with in excess of 10 enteric pathogen-targeting commercial multiplex qPCR kits currently available, including *Allplex, Amplidia, BD Max, Biofire FilmArray, FTD Stool Parasites, Gastro Panel EntericBio Panel II, Gastroenteritis/Parasite Panel I, Luminex, NanoChip, PARAGenie, RIDAGENE,* and *QIAstat* (Table 2) (Autier et al., 2020; Boers et al., 2020; Morio et al., 2019; Hannet et al., 2019; O'Leary et al., 2018; Ryan et al., 2017; Paulos et al., 2019). In addition, multiplex assays significantly reduce reagent and labour costs, and outperform the majority of alternative methods currently in use (Haque et al., 2007; Stark et al., 2011; ten Hove et al., 2007; Verweij and Van Lieshout, 2011). qPCR also readily accommodates genotyping *via* melting curve analysis, and more recently high resolution melting (HRM) curve analysis (Chelbi et al., 2018; Lalonde et al., 2013; O'Leary et al., 2021a, O'Leary et al., 2021b).

While appearing to offer a myriad of diagnostic and logistical advantages, conventional PCR and qPCR have not yet been widely incorporated into routine *Cryptosporidium* detection procedures. This is likely to be due, at least in part, to the requirement for significant investment in reagents and analysers, coupled with extensive personnel training (Burnet et al., 2013; Checkley et al., 2014). However, a future molecular shift in the field of diagnostic parasitology would appear to be inevitable in order to provide essential improvement to current diagnostic services. Thus, the potential benefits must be carefully weighed against the perceived disadvantages associated with molecular methods in the context of current diagnostic limitations.

### 2.4 Future trends in molecular platforms

In recent years a third-generation implementation of conventional PCR that obfuscates the need for calibration curves in the quantification of nucleic acid targets, digital PCR (dPCR), has been gaining traction for its utility in pathogen detection. The method, which is based on the principle of amplifying a single DNA template from maximally diluted samples, therefore generating amplicons that are exclusively derived from one template, based on Poisson statistics, remains a relatively new concept in the field of medical parasitology (Pomari et al., 2019). Unlike qPCR, which produces an exponential signal, dPCR generates linear, digital signals, allowing quantitative analysis of the PCR product, detecting very rare mutations with unprecedented precision and sensitivity (Pohl and Shih, 2004). As of yet, application of dPCR to the detection and quantification of *Cryptosporidium* in human faecal samples is limited to a single study (Yang et al., 2014). However, despite this, the reported precision of dPCR is consistently superior to that of qPCR, and the quantitative detection less affected by the presence of inhibitors. Thus, the initial application of dPCR to the detection and quantification of *Cryptosporidium* oocysts by Yang et al. (2014) may herald its incorporation into mainstream clinical diagnostic methodologies over the coming years.

The emergence and increasing prevalence of next generation sequencing (NGS) technologies is also likely to shape the field of protozoan parasitology over the course of the coming decade, (DeMone et al., 2020). Evaluations of *Cryptosporidium* isolates with NGS techniques have revealed unprecedented within-isolate genetic diversity to a degree that is not possible to discern through current conventional PCR and Sanger sequencing-based subtyping methodologies, given their inability to resolve complex DNA mixtures and detect low-abundance intra-isolate variants (Grinberg et al., 2013; Grinberg and Widmer, 2016; Zahedi et al., 2017). Consequently, NGS-based studies have already advanced current knowledge on the taxonomic distribution and transmission dynamics of *Cryptosporidium* spp., and may also play a future role in outbreak identification and surveillance of new and virulent subtypes (Zahedi et al., 2018; Zahedi et al., 2017).

Despite difficulties in extracting high quality pure DNA from clinical samples, issues with uneven depth of read coverage that leads to gaps in the assembled genome sequence, all of which impact cost and may preclude the widespread use of NGS platforms in clinical laboratories and Public Health agencies; NGS remains likely to exert an important, indirect impact on clinical diagnostics through informing the development of much needed multi-locus sequence typing (MLST) schemes (Cacciò and Chalmers, 2016; Morris et al., 2019). Furthermore, NGS will also undoubtedly play an increasingly pivotal role in epidemiological analyses of *Cryptosporidium* spp., in addition to vaccine and drug development, over the coming decade (Morris et al., 2019; Zahedi et al., 2017).

It would be imprudent to ignore the role of the rapidly-developing fields of bioinformatics and proteomics in this post-genomic era. Efforts to integrate biochemical and genomic data have led to the development of predictive computational models, known as GEMs (genome scale models), for a number of microorganisms in recent years. These models utilise genomic and environmentally based parameters to predict phenotypic outcomes and growth based on biochemical mechanisms (Monk and Palsson, 2014). This is a concept that has already been put into practice for well characterised microorganisms, such as *E. coli* (Carrera et al., 2014)*,* while attempts to develop a genome scale metabolic model of *C. hominis* have already been reported (Vanee et al., 2010). Given the fact that the genomes of both *C. parvum* and *C. hominis* have been fully sequenced, and in light of the studies carried out by Vanee et al. (2010), it is conceivable that GEMs may soon be employed to predict the phenotypic outcome of genetic variations in *Cryptosporidium* species (Vanee et al., 2010; Vinayak et al., 2015).

## 3 Treatment limitations, propagation *via* cell culture, and implications for future advancements

To date, *Cryptosporidium* has remained a largely enigmatic pathogen, owing to its limited tractability and the difficultly encountered in successfully propagating the parasite *in vitro* in cell lines (Karanis and Aldeyarbi, 2011). *In vitro* culture efforts generally result in low yields of mature parasites, as current *Cryptosporidium* cell culture methods generally suffer from rapid host cell overgrowth and ageing, resulting in premature termination of the *Cryptosporidium* lifecycle (Hijjawi, 2010). Normal intestinal epithelial cell (IEC) models fail to adequately recapitulate human intestinal structure and function. Support of parasitic infection by these and immortalised adenocarcinoma derived human IEC models is generally limited to only a few days, precluding parasitic life-cycle completion or continuous propagation (Bhalchandra et al., 2018).

Lack of suitably facile animal models, and molecular tools have also hampered progress in key areas such as developmental biology, the elucidation of host-parasite interactions, biochemical, immunological and molecular studies development, and, perhaps most significantly, evaluation and development of effective anti-cryptosporidial drug therapies (Di Cristina and Carruthers, 2018; Hijjawi et al., 2001). Nitazoxanide, a broad spectrum antimicrobial agent, is currently the only FDA approved treatment for cryptosporidiosis in patients 1 year and older (Sparks et al., 2015). Three double-blind placebo controlled studies suggest the efficacy of nitazoxanide in immunocompetent patients (Amadi et al., 2002; Rossignol et al., 2001, Rossignol et al., 2006). Nitazoxanide, however, is not without considerable limitations in its utility among patient populations that are most vulnerable to *Cryptosporidium* infection. A study conducted on malnourished children found nitazoxanide to improve diarrhoea and morality rates, but the response rate was limited to 56% of studied patients (Amadi et al., 2002). Nitazoxanide has also been found to be ineffective in AIDS patients (Amadi et al., 2009), while various other drugs such as paromomycin, azithromycin, rifamycin, and HIV protease inhibitors have also been unsuccessful in the treatment of cryptosporidiosis in AIDS patients (Checkley et al., 2014; Sparks et al., 2015).

The pursuit of an optimal *in vitro* culture system for *Cryptosporidium* has spanned four decades, with the first complete development of *C. parvum* reported in human, and porcine cell line models in 1984 (Current and Haynes, 1984). In recent years a variety of both cell-free and axenic culture-based systems have yielded promising results. Key milestones in the drive to develop a suitable cell culture-based *in vitro* model have also been published in the past decade (Alcantara Warren et al., 2008; Castellanos-Gonzalez et al., 2008). More recent advancements have sought to extend periods of culture survival (Castellanos-Gonzalez et al., 2013; Jossé et al., 2019; Karanis, 2018; Miller et al., 2018; Varughese et al., 2014). Hollow fibre technologies and stem-cell derived organoids, have also provided a basis for sustained *in-vitro* oocyst propagation (Morada et al., 2016). The development of a system employing stem-cell derived small intestinal, and lung organoids, capable of recapitulating the *in vivo* physiology of their original tissues, to model *Cryptosporidium* infection has been described. These organoids support propagation and completion of the parasitic life-cycle, generating infectious oocysts equivalent to those derived from animal models (Dutta et al., 2019; Heo et al., 2018).

Advancements in cell culturing methodologies have enabled several compound screening studies to be conducted. These screening studies have yielded promising potential anti-cryptosporidial compounds. A HCT-8 cell-based, high-throughput screen (HTS) of the Medicines for Malaria (MMV) Open Access Malaria box, a collection of 400 compounds selected from 19,000 structurally unique molecules that were shown to have activity against the *Plasmodium falciparum*, was conducted by Bessoff et al. (2014) and identified several scaffold structures with activity against *C. parvum*. Another study screened a bank of over 6,000 compounds known to exhibit anti-protozoan activity *via* a high-content imaging infection assay in HCT-8 cells resulting in the identification of the *Cryptosporidium* orthologue to *Plasmodium* lipid kinase PI(4)K, *Cryptosporidium* lipid kinase PI(4)K, as a potential target for pyrazolopyridines based therapeutic candidates (Manjunatha et al., 2017).

Most significantly, however, is the recent application of the CRISPR/Cas system genome editing technology to *Cryptosporidium*. In a major breakthrough for *Cryptosporidium in vitro* research, Vinayak et al. (2015) harnessed the CRISPR/Cas system, to successfully transfect *C. parvum* sprozoites in tissue culture and subsequently isolate genetically modified *C. parvum* sporozoites. Such a demonstration of genetically modified *Cryptosporidium* sporozoites was the first of its kind, paving the way for the development of subsequent parasite survival assays that are furthering contemporary understanding of the basic biology and virulence of this pathogen, and tractable *in vivo* models (Pawlowic et al., 2019; Sateriale et al., 2019; Vinayak et al., 2015).

Similar to the advances made in NGS technologies, future applications of CRISPR/Cas systems may result in far reaching implications and advancements in diverse areas of *Cryptosporidium* research. For example, given the severe limitations associated with current treatment options, the recent advancements employing the aforementioned CRISPR/Cas9 genetic modification of *C. parvum* may prove critical in the development of vaccines and more effective drug therapies, particularly given the utility of gene ablation in identifying alternative treatment strategies (Doudna and Charpentier, 2014; Vinayak et al., 2015). Additionally, the recent incorporation of the CRISPR/Cas12a system within a fluorescent lateral flow strip biosensor tailored for on-site diagnosis of *Cryptosporidium parvum* subtype family IId demonstrates the clinical potential and diagnostic utility of CRISPR/Cas system in *Cryptosporidium* detection (Yu et al., 2021).

## 4 Concluding remarks

The 2015 Nobel Prize in Physiology or Medicine is a pertinent reminder of the importance and necessity for continued research in the field of parasitology. The work of Tu Youyou and the combined efforts of William C. Campbell and Satoshi Ōmura were recognised for the discoveries made concerning the development of novel therapies for malaria, a parasite also of the apicomplexan phylum, and roundworm, respectively (Długońska, 2015). Such high profile recognition in the field of parasitology is arguably suggestive of a resurgence of interest in the field, which has stagnated somewhat in terms of clinical, therapeutic and molecular diagnostic advances. The renaissance of *Cryptosporidium* research is already underway; the major breakthrough in producing CRISPR/Cas modified, tractable *C. parvum* oocysts signifies a reinvigoration of the field. This advancement is likely aid the advancement of knowledge in areas such as host-parasite interactions, and the biochemical and immunological pathways at play within *Cryptosporidium* spp. It will also facilitate the identification and validation of much needed drug targets.

It is also crucial to note that the causative agent remains undetected in 70% of gastroenteritis cases. Advances in diagnostic approaches for *Cryptosporidium* spp., and parasitic species in general, may play a significant role in further elucidating the nature of this unknown, and potentially diverse, pathogenic conglomerate (Freedman et al., 2015). This is particularly relevant in light of the human and economic toll exerted by gastrointestinal infection on a global scale, which according to a recent WHO study affected 2 billion people, caused 1 billion deaths and 78.7 million disability adjusted life years in 2010 (Kirk et al., 2015). As a consequence, *Cryptosporidium* research has perhaps never been more pertinent than it is today.

## Funding

This work was supported by the 10.13039/501100002081Irish Research Council [GOIPG/2014/918].

## Declaration of Competing Interest

There are no conflicts of interest, of which we are aware, relating to this body of work.
